# Transcriptional Networks Identify BRPF1 as a Potential Drug Target Based on Inflammatory Signature in Primary Lower-Grade Gliomas

**DOI:** 10.3389/fonc.2021.766656

**Published:** 2021-12-02

**Authors:** Mingyang Xia, Huiyao Chen, Tong Chen, Ping Xue, Xinran Dong, Yifeng Lin, Duan Ma, Wenhao Zhou, Wei Shi, Hao Li

**Affiliations:** ^1^ Key Laboratory of Birth Defects, Children’s Hospital of Fudan University, Shanghai, China; ^2^ Center for Molecular Medicine, Children’s Hospital of Fudan University, Institutes of Biomedical Sciences, Fudan University, Shanghai, China; ^3^ Department of Neurosurgery, Children’s Hospital of Fudan University, Shanghai, China; ^4^ Center for Molecular Medicine, Children’s Hospital of Fudan University, Shanghai, China; ^5^ Key Laboratory of Neonatal Diseases, Division of Neonatology, Children’s Hospital of Fudan University, Ministry of Health, Shanghai, China; ^6^ Key Laboratory of Metabolism and Molecular Medicine, Ministry of Education, Department of Biochemistry and Molecular Biology, School of Basic Medical Sciences and Institutes of Biomedical Sciences, Shanghai Medical College, Fudan University, Shanghai, China

**Keywords:** lower-grade gliomas, inflammatory signature, prognostic marker, drug targets, BRPF1

## Abstract

Gliomas are the most common tumors of the central nervous system and are classified into grades I-IV based on their histological characteristics. Lower-grade gliomas (LGG) can be divided into grade II diffuse low-grade gliomas and grade III moderate gliomas and have a relatively good prognosis. However, LGG often develops into high-grade glioma within a few years. This study aimed to construct and identify the prognostic value of an inflammatory signature and discover potential drug targets for primary LGG. We first screened differentially expressed genes in primary LGG (TCGA) compared with normal brain tissue (GTEx) that overlapped with inflammation-related genes from MSigDB. After survival analysis, nine genes were selected to construct an inflammatory signature. LGG patients with a high inflammatory signature score had a poor prognosis, and the inflammatory signature was a strong independent prognostic factor in both the training cohort (TCGA) and validation cohort (CGGA). Compared with the low-inflammatory signature group, differentially expressed genes in the high-inflammatory signature group were mainly enriched in immune-related signaling pathways, which is consistent with the distribution of immune cells in the high- and low-inflammatory signature groups. Integrating driver genes, upregulated genes and drug targets data, bromodomain and PHD finger-containing protein 1 (BRPF1) was selected as a potential drug target. Inhibition of BRPF1 function or knockdown of BRPF1 expression attenuated glioma cell proliferation and colony formation.

## Introduction

Gliomas are heterogeneous brain tumors with a poor prognosis derived from glial cells (1). According to their histological characteristics, gliomas are classified as grade I-IV by the World Health Organization (WHO) (2). Lower-grade gliomas (LGG) are comprised of grade II diffuse low-grade gliomas and grade III moderate gliomas, which is also consistent with the classification in the TCGA datasets, including astrocytoma, oligoastrocytoma and oligodendroglioma, accounting for 15-20% of all gliomas, and their median survival time is approximately 10 years (1, 3–6). Currently, maximum surgical resection combined with postoperative chemotherapy and radiotherapy is the main treatment for LGG (7).

1p/19q codeletion, MGMT promoter methylation and IDH mutations often occur in gliomas and are associated with their prognosis. These biomarkers were integrated into the 2016 WHO CNS classification to illustrate their histological features and guide clinical therapy (2, 8–11). Nonetheless, some LGG still progress to high-grade glioma within a few years after molecular diagnosis and conventional treatment (12). Therefore, new prognostic biomarkers are needed to better predict the clinical outcomes of LGG patients and tailor therapeutic strategies.

Inflammation is a physiological response caused by trauma, chemical irritation/injury, or infection (13, 14). It is also related to cancer development, involving genotoxicity, abnormal tissue repair, proliferative response, invasion and metastasis (15–18). The inflammatory signaling pathway plays an important role in carcinogenesis, such as the STAT3 and NF-κB signaling pathways (16). In glioma, cytokine-mediated inflammation cascades contribute to angiogenesis, tumor growth and metastasis. The inflammatory microenvironment also has an immunosuppressive effect, which impedes the success of various glioma immunotherapies (19, 20). Previous studies on inflammatory response biomarkers have mainly focused on the neutrophil-to-lymphocyte ratio (NLR), platelet-to-lymphocyte ratio (PLR) or lymphocyte-to-monocyte ratio (LMR) to evaluate the prognosis of patients with cancer. Patients with high NLR and PLR and low LMR have a worse prognosis in a variety of tumors (21–24). During tumorigenesis and progression, inflammatory response-related gene expression is often altered (15–17). However, the clinical prognostic effect of the inflammatory signature based on inflammatory response gene expression in lower-grade gliomas remains unclear.

In this study, we analyzed differentially expressed genes (DEGs) between primary LGG tissue (TCGA) and normal brain tissue (GTEx). Then, we obtained the overlapping genes between differentially expressed genes (DEGs) and inflammatory response genes (IRGs) from MSigDB to construct an inflammatory signature for primary LGG outcome prediction in the training cohort (TCGA) and validation cohort (CGGA cohort 1 and CGGA cohort 2). A nomogram was also constructed based on the multivariate Cox regression analysis results that integrated the inflammatory signature and clinicopathological features. Finally, we analyzed the immune cell landscape and transcriptional characteristics between the high- and low-inflammatory signature groups and identified BRPF1, which is related to the proliferation of glioma cells, as a potential drug target.

## Materials and Methods

### Patient Datasets

The RNA-sequencing data of 504 patients with primary LGG from the TCGA as the training cohort and 725 normal brain samples from GTEx were obtained from the University of California Santa Cruz (UCSC) Xena website (https://xena.ucsc.edu). The RNA-sequencing data of 407 patients (including 270 patients in CGGA cohort 1 and 137 patients in CGGA cohort 2) were downloaded from the CGGA (http://www.cgga.org.cn) as the validation cohort. Corresponding clinical information of patients in the training cohort and validation cohort was acquired from the TCGA and CGGA, respectively.

### Differential Expression Analysis

Gene expression was quantified by normalized estimation of fragments per thousand base transcripts per million mapped reads (FPKM) and log2-based transformation. The ComBat method was performed to remove the batch effects using the R package “sva”. Next, DEGs were identified by the “limma” package in R software using the absolute value of the log2-transformed fold change (FC) > 2 and the adjusted P value (adj. P) < 0.05 as the threshold.

### Construction of the Inflammatory Signature

Univariate Cox regression analysis was performed to analyze the prognostic significance of overlapping genes between the DEGs and IRGs. Twenty-seven genes correlated with overall survival (OS) (P < 0.05) were screened out. Furthermore, nine genes and their regression coefficients obtained by least absolute shrinkage and selection operator (LASSO, R package: glmnet) regression analysis were applied to construct the inflammatory signature. A 10-fold cross-validation was performed to select the optimal lambda (penalty for the number of characteristics), which determined the performance of the lasso-cox model (number of features included in the model and predictive deviations). The inflammatory signature score of each patient in the training cohort and validation cohort was calculated by the following formula:


$$Inflammatory\;signature\;score=\sum_{n=1}^{n}\left(\beta_{n}\times x_{n}\right)$$

*β_n_* is the coefficient of each gene derived from the LASSO regression, and *x*_n_ is the expression level of each gene. Primary LGG patients were divided into high- and low-inflammatory signature groups in the training cohort (TCGA) and validation cohort (CGGA) according to the median inflammatory signature score.

### Functional Annotation, Enrichment Analysis and Construction of the Protein-Protein Interaction (PPI) Network

Gene set enrichment analysis (GSEA), gene set variation analysis (GSVA), gene ontology (GO) and Kyoto Encyclopedia of Genes and Genomes (KEGG) analyses were applied to explore the biological functions and signaling pathways related to the high- and low-inflammatory signature groups using the MSigDB database. Gene sets with adjusted P values <0.05 were included in the analysis. The STRING database (https://string-db.org/) was used to construct a PPI network of overlapping genes between the DEGs and IRGs. The PPI network was visualized using the Cytoscape software.

### NetBID Algorithm

We integrated the TCGA-LGG gene expression profile and computationally reconstructed a brain-specific transcriptional network using the SJARACNe algorithm (25). The network included potential master regulators (1899 transcription factors and 8403 signaling proteins) with their transcriptionally predicted target genes. Then, we used the network-based Bayesian inference of drivers (NetBID) (26) (https://github.com/jyyulab/NetBID) algorithm to infer the regulatory activity of the master regulators in each sample based on their target gene expression value and the regulatory relationship. We hypothesized that if a transcription factor/signaling protein is a “hidden” driver between LGG subsets, its regulons in the network should be enriched in the differentially expressed genes, although the driver itself is not necessarily differentially expressed.

### Cell Culture

Human glioma cell lines (U87-MG and U251) were acquired from the Cell Bank of the Chinese Academy of Sciences (Shanghai, China), cultured in DMEM (Gibco, USA) supplemented with 10% fetal bovine serum (Gibco, USA) and penicillin-streptomycin-glutamine (Gibco, USA) and incubated at 37°C in a 5% CO_2_ humidified atmosphere.

### Cell Viability Assay

The viability of human glioma cell lines (U87-MG and U251) treated with BRPF1-specific inhibitor (GSK6853, Selleck) or vehicle (DMSO, Sigma) was measured by Cell Counting Kit-8 (CCK-8) according to the manufacturer’s protocol. Briefly, U87-MG and U251 cells (2 × 10^3^/well) were seeded into 96-well cell culture plates and incubated with a BRPF1-specific inhibitor (GSK6853, Selleck) or vehicle (DMSO, Sigma) for 24-96 h at 37°C in a 5% CO_2_ humidified atmosphere. CCK-8 reagent was added to the medium and incubated for 1-2 h at 37°C in a 5% CO_2_ humidified atmosphere. Cell culture plates were read at 450 nm, and the OD value was obtained. Cell proliferation curves were produced using GraphPad Prism 8.

### Western Blot Analysis

Total protein of human glioma cell lines (U87-MG and U251) was extracted with RIPA lysis buffer (Millipore, Cat. No: 20-188) containing protease inhibitor (Roche, Cat. No: 11873580001) and quantified *via* BCA assay (Beyotime, Cat. No: P0012S). 10% SDS-gel were applied to separate proteins. Primary antibodies against BRPF1 (Abcam, Cat. No: ab251669,1:1000 dilution) and β-actin (ORIGENE, Cat. No: TA811000, 1:2000 dilution) were used for immunoblotting. Enhanced chemiluminescence was used to detect protein bands, and the intensity of the protein bands was determined with an Image software (Bio-Rad).

### Construction of BRPF1 Knockdown Plasmid

The pGreenPuro (CMV) vector was used to construct a BRPF1 knockdown plasmid. The shRNA sequences were as follows: shBRPF1-1#, AGGACTACATCTGGCTGGATATCAT, and shBRPF1-2#, CCGCATCAGCATCTTTGACAA.

### Soft-Agar Colony Formation Assay

One milliliter of DMEM containing 10% FBS with 0.6% agarose (Sangon Biotech) was added to a 12-well cell culture plate as a base support. Human glioma cell lines (U87-MG and U251) were seeded in 1 ml of DMEM containing 10% FBS with 0.35% agar at 1 × 10^4^ cells/well and layered onto the base support. Then, 0.5 ml of DMEM containing 10% FBS with BRPF1-specific inhibitor (GSK6853, Selleck) or vehicle (DMSO, Sigma) was layered on top of the agar gel. Three weeks later, the number of colonies in each well was counted under a microscope (Leica DMIL LED).

### Statistical Analysis

All statistical data are shown as the mean ± standard deviation (SD). The Student’s t-test or analysis of variance (ANOVA) was used for statistical analyses. Survival analyses were compared between the high- and low-inflammatory signature groups *via* Kaplan-Meier analysis methods using the ‘survival’ and “survminer” packages in R. Univariate Cox regression analysis was applied to identify potential prognostic genes, and Lasso regression was performed to screen out gene sets to construct an inflammatory signature. Multivariate Cox regression analysis was used to determine clinical factors (including the inflammatory signature) as independent risk factors for OS in primary LGG. The methodology of Grambsch and Therneau was used to verify the proportional hazards assumption in the Cox proportional hazards model. A P value < 0.05 was defined as statistically significant.

## Results

### Patient Characteristics

**Figure 1:**
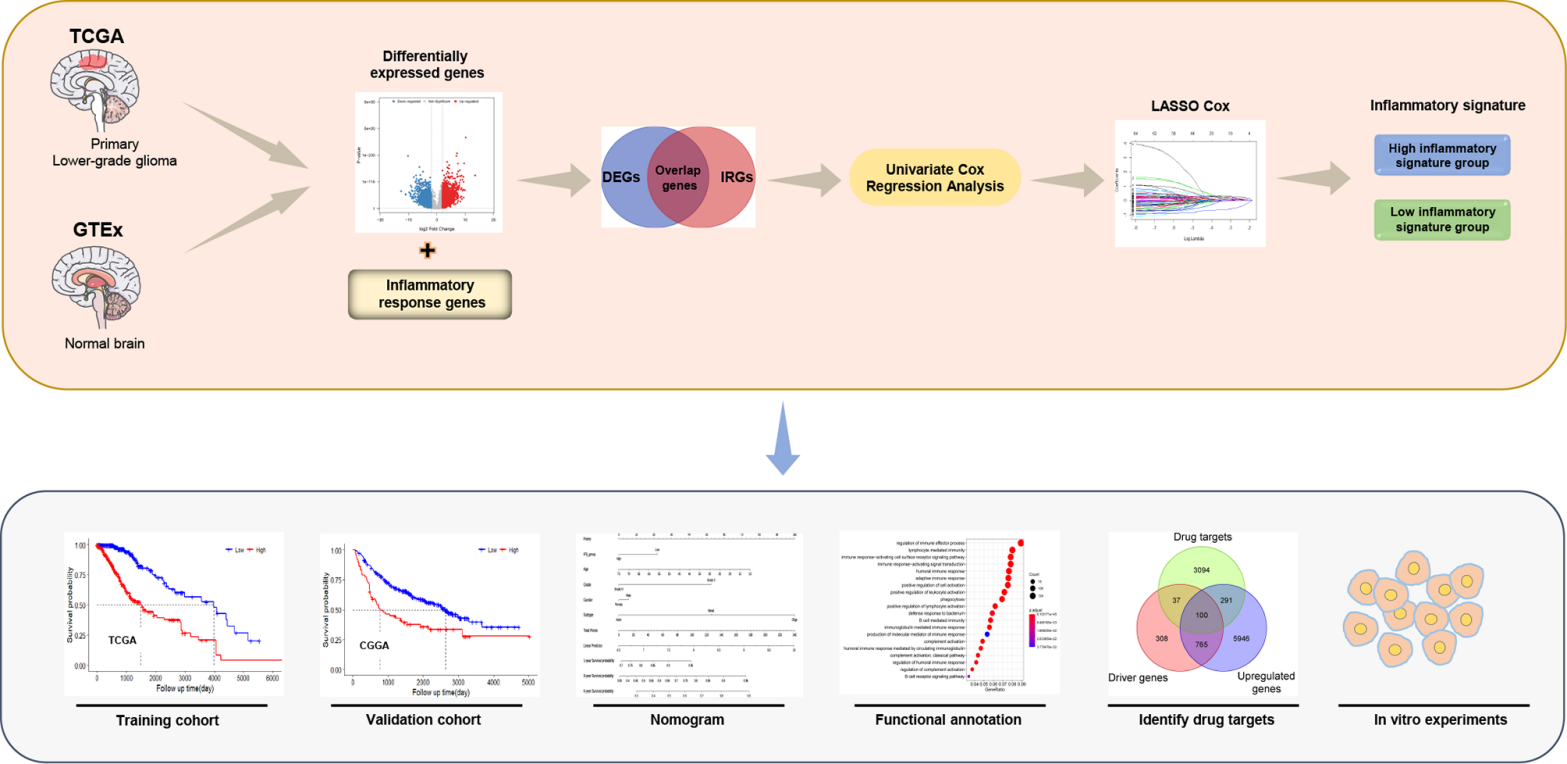
The flowchart of this study.

The workflow of our study is presented in **Figure 1**. A total of 911 patients with primary LGG met the inclusion criteria, including 504 patients from the TCGA as the training cohort and 407 patients from the CGGA as the validation cohort (270 patients in CGGA cohort 1 and 137 patients in CGGA cohort 2). The clinical characteristics of the primary LGG patients from the TCGA and CGGA are listed in **Table 1**.

**Table 1:** Clinical characteristics of the primary lower-grade gliomas.

Clinical characteristic		TCGA	CGGA	
		n = 504	cohort 1 (n = 270)	cohort 2 (n = 137)
Age	< 60	443	260	126
	≥60	61	10	11
Gender	Male	281	150	85
	Female	223	120	52
Grade	II	244	130	90
	III	259	140	47
	Unknow	1	0	0
Histology subtype	Oligodendroglioma	185	89	56
	Astrocytoma	192	158	81
	Mixed glioma	127	23	0
Radiation therapy	Yes	270	199	120
	No	168	67	13
	Unknow	66	4	4
Chemical therapy	Yes	–	170	65
	No	–	94	64
	Unknow	–	6	8
IDH mutation	Yes	89	176	101
	No	34	64	35
	Unknow	381	30	1
1p19q_codeletion_status	Codel	–	81	50
	Non-codel	–	156	85
	Unknow	–	33	2
MGMT methylation	Methylated	–	127	64
	Un-methylated	–	88	61
	Unknow	–	55	12

IDH, isocitrate dehydrogenase; MGMT, O6-methylguanine-DNA methyltransferase; -, not reported.

### Identification of Inflammation-Related Genes With Prognostic Significance

**Figure 2:**
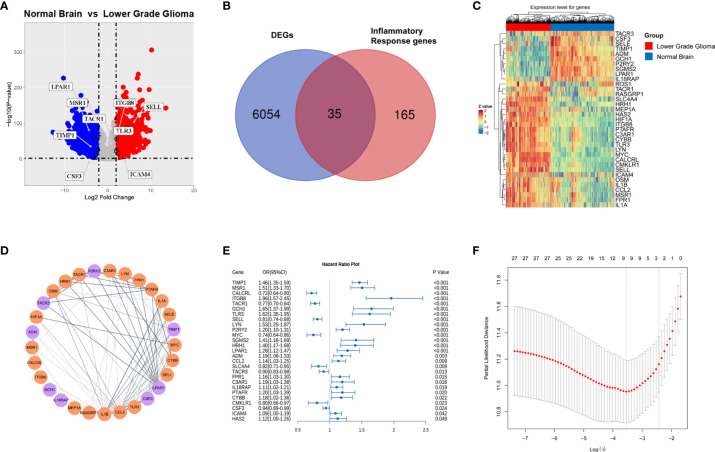
Identification of inflammation-related genes with prognostic significance.**(A)** Volcano plot of differentially expressed genes analysis in primary lower grade glioma (TCGA cohort) compared with normal brain (GTEx) (red dots, upregulated genes; blue dots, downregulated genes); **(B)** Venn diagram of overlapping genes between the DEGs and IRGs; Gene expression heatmap **(C)** and PPI network **(D)** of overlapping genes. **(E)** Forest plot of overlapping genes correlated with survival in the TCGA cohort (p value <0.05). **(F)** Cross-validation for tuning parameter (lambda) screening in the lasso regression model.

To identify inflammation-related genes with prognostic significance, we first downloaded RNA-seq data of normal brain tissue from GTEx and primary LGG from the TCGA. After normalization and batch effect removal, a total of 6,089 DEGs were selected using the absolute value of the log2-transformed fold change (FC) > 2 and the adjusted P value (adj. P) < 0.05 as the threshold (**Supplementary Table S1**). Among them, 3734 genes were upregulated and 2355 genes were downregulated, as shown in **Figure 2A**. Two hundred IRGs were downloaded from MSigDB. After overlapping the DEGs and IRGs, thirty-five genes were obtained (**Figure 2B**), and 71.34% of these genes (25/35) were upregulated in the LGG group (**Figure 2C**). STRING was used to construct the PPI network of overlapping genes. After visualization using the Cytoscape software, we discovered that some proteins were closely related to other proteins, such as LPAR1, IL1β, CCL2, MYC, and IL1α (**Figure 2D**).

Next, thirty-five overlapping genes were further analyzed by univariate Cox regression analysis, and twenty-seven genes significantly associated with OS in primary LGG (TCGA) were obtained (**Figure 2E**). Considering collinearity, twenty-seven inflammatory prognostic genes were subjected to Lasso Cox regression, and nine genes, including CSF, SELL, TACR1, ICAM4, ITGB8, LPAR1, MSR1, TLR3 and TIMP1, were obtained to construct an inflammatory signature (**Figure 2F**). Nine pivotal genes illustrated the differences in the survival time of patients with primary LGG (TCGA). CSF, SELL and TACR1 were correlated with an adverse prognosis, while the other six genes were correlated with a good prognosis (**Figure S1**).

### Establishment and Validation of the Inflammatory Signature

**Figure 3:**
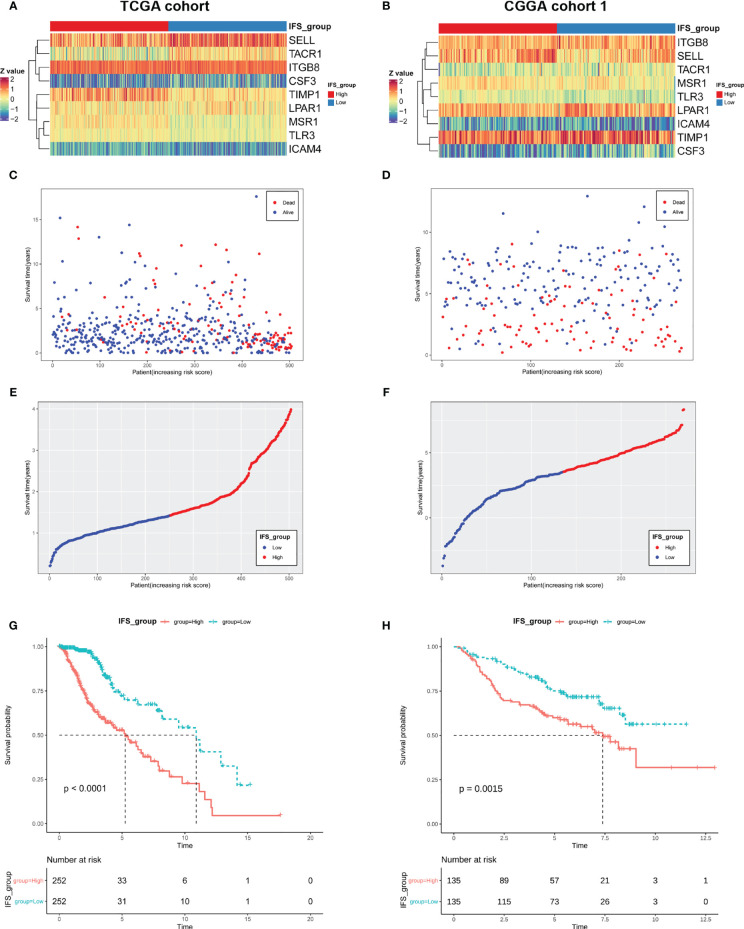
LGG patients with a high inflammatory signature had a worse prognosis in the TCGA cohort and CGGA cohort 1. Gene expression heatmap of the nine inflammation-related genes with prognostic significance in the TCGA cohort **(A)** and CGGA cohort 1 **(B)**. Survival status diagram of LGG patients in the TCGA cohort **(C)** and CGGA cohort 1 **(D)** (red dots represent death, and blue dots represent survival). Score distribution diagram of LGG patients with high- or low-inflammatory signature groups in the TCGA cohort **(E)** and CGGA cohort 1 **(F)** (red: high IFS; blue: low IFS). Survival curve for LGG patients with high- or low-inflammatory signature groups in the TCGA cohort **(G)** and CGGA cohort 1 **(H)**.

An inflammatory signature (IFS) was constructed with nine genes,and their coefficients were previously identified. The gene expression profile of the nine genes in the training cohort (TCGA) is shown in **Figure 3A**. The primary LGG patients in the training cohort (TCGA) were divided into high- and low-inflammatory signature groups using the median inflammatory signature score as the cutoff value. The inflammatory signature score and survival status distribution of the primary LGG patients in the training cohort (TCGA) are shown in **Figures 3C, E**. The Kaplan-Meier survival curve showed that patients in the high-inflammatory signature group (high IFS group) had shorter survival time (P< 0.0001; **Figure 3G**).

The 270 primary LGG patients from the CGGA were used as a validation cohort 1 to verify the performance of the inflammatory signature. The inflammatory signature score for each patient in the validation cohort 1 (CGGA cohort 1) was calculated using the same method. The primary LGG patients in the CGGA cohort 1 were also divided into high- and low-inflammatory signature groups using the median inflammatory signature score. The gene expression profile of the nine genes, inflammatory signature score and survival status distribution of the primary LGG patients in the CGGA cohort 1 are shown (**Figures 3B, D** **, F**). We found that primary LGG patients in the high-inflammatory signature group had a worse prognosis (P=0.0015; **Figure 3H**). Consistently, the similar results were discovered in validation cohort 2 (CGGA cohort 2) (**Figure S2**). These results suggested that the inflammatory signature was a good predictor of the OS of patients with primary LGG.

### Independent Predictive Ability of the Inflammatory Signature in the TCGA and CGGA

**Figure 4:**
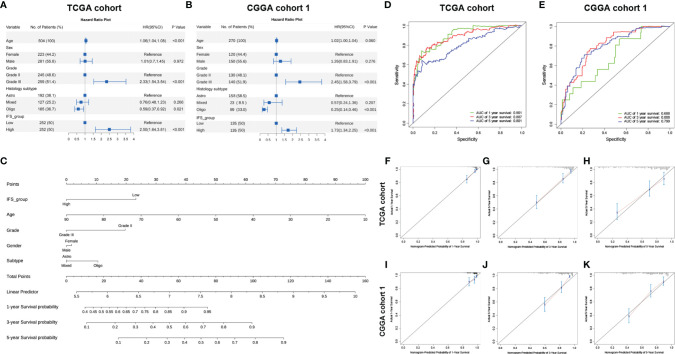
The inflammatory signature was a strong independent prognostic factor for LGG patients in the TCGA cohort and CGGA cohort 1. Forest plot of multivariate regression analysis in the TCGA cohort **(A)** and CGGA cohort 1 **(B)**. Nomogram based on the results of multivariate cox regression analysis in the TCGA cohort **(C)**. The ROC curve and AUC of the predictions for 1, 3, and 5 years of the nomogram for the TCGA cohort **(D)** and CGGA cohort 1 **(E)**. The calibration curves for predicting the 1-, 3-, and 5-year survival in the TCGA cohort **(F–H)** and CGGA cohort 1 **(I–K)**.

To assess the independent prognostic role of the inflammatory signature, univariate and multivariate Cox regression analyses were applied to confirm its performance in the training and validation cohorts. The results from univariate Cox regression analysis showed that IFS_group, grade, histology subtype, age in the training cohort and grade and histology subtype in the validation cohort 1 and IFS_group, grade, histology subtype, age in the validation cohort 2 were significantly associated with patient survival (**Figures S3** and **S4A**). Multivariate Cox regression analysis showed that the high-inflammatory signature group was independently associated with a worse OS of primary LGG patients in both the training and validation cohort 1 (P<0.001; **Figures 4A, B**). These results indicated that the inflammatory signature was a strong independent prognostic factor for patients with primary lower-grade gliomas.

Based on the multivariate Cox regression analysis, a nomogram was constructed for predicting primary LGG 1-, 3- and 5-year OS time, which integrated both the inflammatory signature group and clinicopathologic variables, including age, sex, histological subtype, and grade (**Figure 4C**). The data analysis conformed to the proportional hazards assumption (TCGA: P = 0.062, CGGA cohort 1: P = 0.099, CGGA cohort 2: P =0.621). The C-index of the nomogram in the training cohort (TCGA) was 0.826 (95% CI; 0.787–0.865). The areas under the curve (AUC) of the 1-, 3- and 5-year OS predictions for the constructed nomogram were 0.901, 0.887 and 0.801 in the training cohort, respectively (**Figure 4D**). Meanwhile, calibration curve for this nomogram were developed and plotted, which showed that this nomogram model had good accuracy (**Figures 4F–H**). In the validation cohort (CGGA cohort 1 and CGGA cohort 2), we found consistent results. The areas under the curve (AUC) and calibration curve were also plotted (**Figures 4E, I**–**K** and **S4C**–**F**). These results demonstrated that the nomogram had good accuracy in predicting the 1-, 3- and 5-year survival of patients with primary LGG in both the training cohort (TCGA) and validation cohort (CGGA cohort 1 and CGGA cohort 2).

### Identification of the Immune Cell Landscape and Transcriptional Characteristics Between the High- and Low-Inflammatory Signature Groups

**Figure 5:**
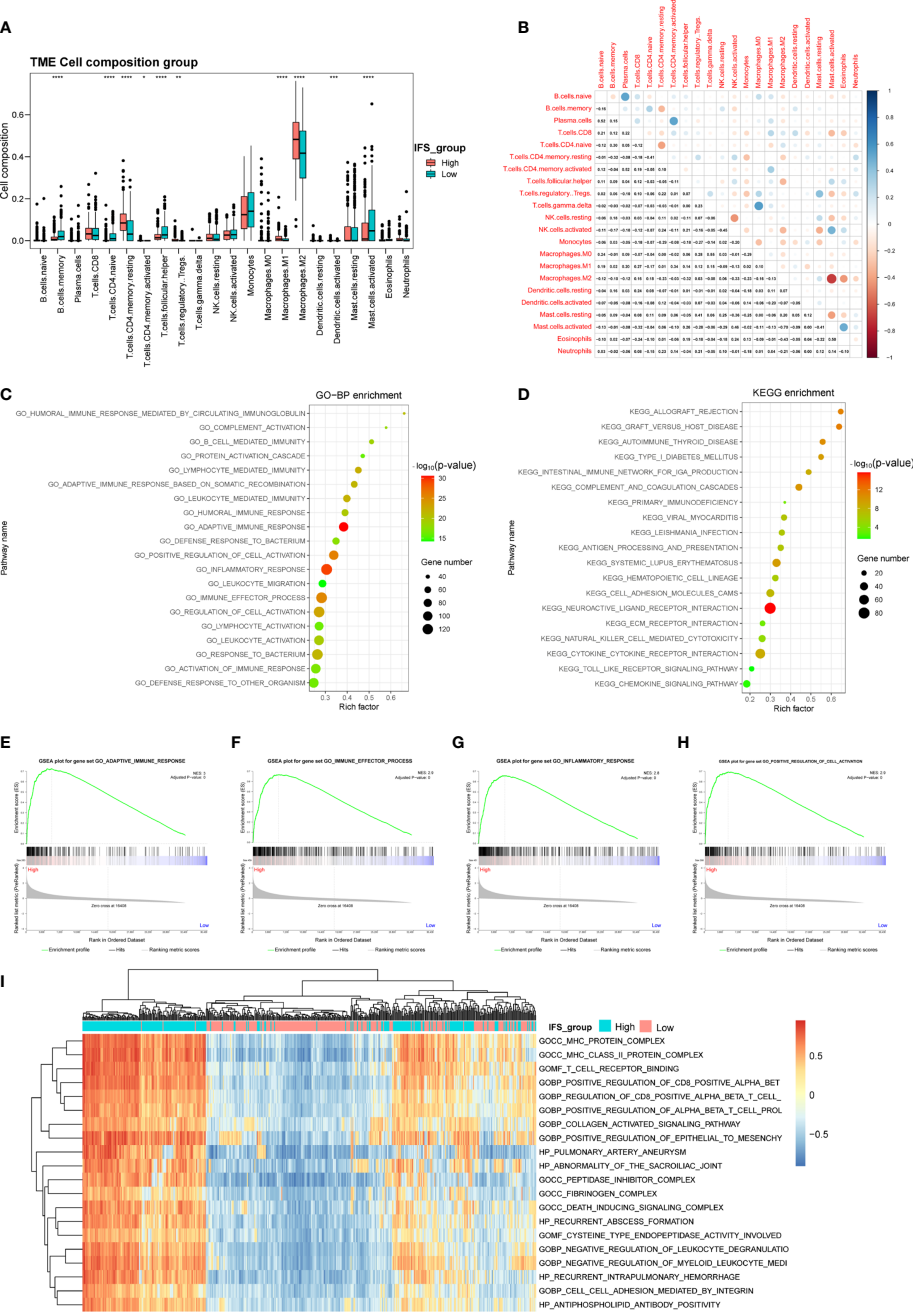
Identification of the immune cell landscape and transcriptional characteristics between the high- and low-inflammatory signature groups in the TCGA cohort. **(A)** Comparison of the immune cell composition between the high- and low-inflammatory signature groups in the TCGA cohort. **(B)** Correlation of 22 types of immune cell subsets in the TCGA cohort. GO **(C)**, KEGG pathway **(D)**, GSEA **(E–H)** and GSVA **(I)** analyses of differentially expressed genes between the high- and low-inflammatory signature groups in the TCGA cohort. *p< 0.05; **p< 0.01; ***p < 0.001; ****p < 0.0001; Wilcoxon test was used to assess the significance of the immune cell composition.

In the disease development process, changes in inflammation levels in patients are often accompanied by an immune response (18). Previous studies have reported that alterations in immune cells in the tumor microenvironment are related to tumorigenesis and progression (27–29). To explore the differences in immune cells in LGG patients with high- and low-inflammatory signature, we adopted the CIBERSORT method to analyze the distribution of immune cells in LGG tissues in the training cohort (TCGA). After deconvolution, M2 macrophages were the most abundant immune cells, followed by monocytes and activated mast cells (**Figure 5A**). Then, we confirmed similar results in the CGGA cohort 1. M2 macrophages were also the most abundant immune cells (**Figure S3A**). The proportions of M2 macrophages and resting CD4 memory T cells in the high-inflammatory signature group were significantly higher than those in the low-inflammatory signature group in the TCGA and CGGA cohort 1 (**Figures 5A**, **S5A**). These results showed the heterogeneity of the immune cells of the TME in primary LGG and M2 macrophages and resting CD4 memory T cells that demonstrated high activity in the TME during the primary LGG development process.

Correlation analysis based on the training cohort (TCGA) suggested that the number of M2 macrophage cells was inversely related to the number of activated mast cells (r^2^= -0.70). Activated NK cells were negatively correlated with resting NK cells (r^2^= -0.45), and naïve CD4 T cells were negatively correlated with resting memory CD4 T cells (r^2^= -0.41) (**Figure 5B**). These results indicated that antagonistic functions might exist between these cells in the LGG development process. In contrast, we found a positive correlation between plasma cells and naïve B cells or activated memory CD4 T cells (r^2 =^ 0.52 or 0.52) in the TCGA cohort and a highly positive correlation between regulatory T cells (Tregs) and resting NK cells or resting mast cells (r^2 =^ 0.54 or 0.42) in the CGGA cohort 1(**Figures 5B**, **S5B**). These results suggest that these cells might have synergistic functions in the tumor microenvironment.

To depict transcriptional characteristics between the high- and low-inflammatory signature groups, the DEGs were further screened by comparing the gene expression profiles. A total of 2123 upregulated genes and 1690 downregulated genes were selected in the high-inflammatory signature group compared with the low-inflammatory signature group in the TCGA cohort with the absolute value of fold change >1 and adjusted P value (adj. P) <0.05 as the threshold (**Supplementary Table S2**). The GO analysis results showed that the differentially expressed genes were mainly enriched in biological processes linked to inflammatory response and immunity, such as adaptive immune response, leukocyte-mediated immunity and immune effector process (**Figure 5C**). The bar plot of the KEGG analysis revealed that immune-related signaling pathways were enriched, such as neuroactive ligand receptor interaction, systemic lupus erythematosus and primary immunodeficiency pathways (**Figure 5D**). GSEA and GSVA were also performed to decipher the difference between the high- and low- inflammatory signature groups. The GSEA and GSVA results were also involved in the immune process (**Figures 5E**–**I**). Interestingly, similar results were found in the CGGA cohort 1. The results of the GO, KEGG, GSEA and GSVA analyses were also enriched for immunity (**Figures S5C**–**I**).

### Screening of Drug Targets Based on the Inflammatory Signature

**Figure 6:**
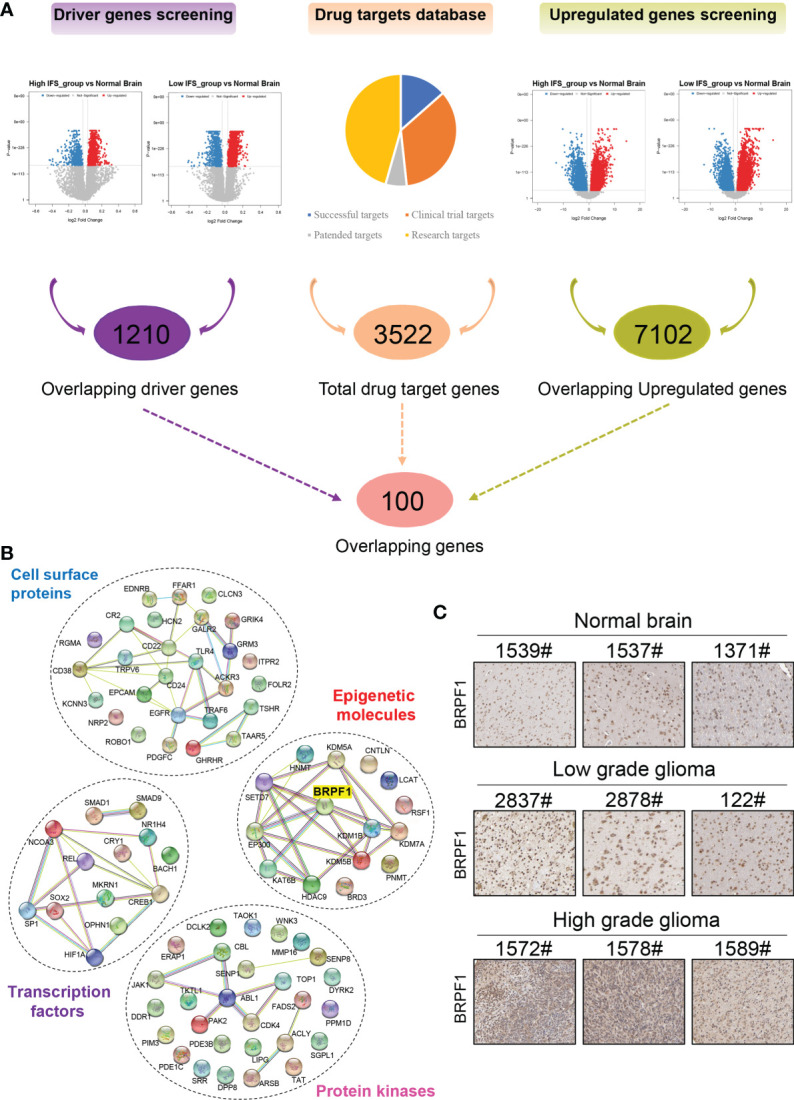
Screening of drug targets based on the inflammatory signature. **(A)** Screening flowchart of potential drug targets based on the transcriptome data between the normal brain and the high- or low-inflammatory signature groups. **(B)** The protein-protein interaction network of the following four categories: transcription factors, epigenetic molecules, protein kinases, and cell surface proteins. **(C)** The protein level of BRPF1 in normal brain, low-grade glioma and high-grade glioma tissues from the Human Protein Atlas.

Maximum surgical resection combined with radiotherapy and chemotherapy is the main treatment protocol for gliomas (7). However, some patients still suffer from surgical sequelae and tumor recurrence. In addition, targeted therapy is relatively rare due to the lack of effective drug targets (30). In our study, a three-step approach was developed to screen drug targets for primary lower-grade gliomas based on the inflammatory signature. First, combined with transcriptomic data in the TCGA cohort, the NetBID algorithm was applied to screen out “hidden” driver genes between the normal brain and high- or low-inflammatory signature groups, and 1210 overlapping genes were obtained (**Supplementary Table S3**). Second, the DEGs between the normal brain and the high- or low-inflammatory signature group were identified by bioinformatics methods, and 7102 overlapping upregulated genes were obtained (**Supplementary Table S4**). Finally, the information of 3522 drug targets in the therapeutic target database (TTD) was integrated, and 100 overlapping genes were identified that could be potential drug targets for lower grade gliomas with high- and low-inflammatory signatures (**Figure 6A** and **Supplementary Table S5**). After gene annotation, the 100 overlapping genes could be mainly classified into the following four categories: transcription factors, epigenetic molecules, protein kinases, and cell surface proteins (**Figure 6B**). In the validation cohort (CGGA cohort 1 and CGGA cohort 2), we obtained 218 and 475 overlapping genes using same screening method, respectively (**Supplementary Tables S6**, **S7**). Finally, we identified 22 common genes among TCGA cohort, CGGA cohort 1 and CGGA cohort 2 (**Supplementary Table S8**).

Epigenetics plays an important role in the pathogenesis of nervous system diseases (including tumors), and several epigenetic regulatory molecules have been considered potential drug targets (31–34). Some studies have reported that epigenetic molecules containing bromodomains can be used as drug targets, such as BRD4 (35), p300/CBP (36), and TRIM24 (37). Drugs or inhibitors that target these molecules, such as JQ1, have good antitumor effects (38, 39). Among the 22 common genes, epigenetic molecule BRPF1 contains bromodomain and has been identified as a therapeutic target for liver cancer (40). Therefore, we selected BRPF1 for further research in this study. According to the Human Protein Atlas database, the protein level of BRPF1 was higher in low- and high-grade gliomas than in normal brain tissue, and the highest expression level of BRPF1 was found in high-grade gliomas (**Figure 6C**).

### Inhibition of BRPF1 Function or Interference of BRPF1 Expression Attenuated Glioma Cell Proliferation and Colony Formation

**Figure 7:**
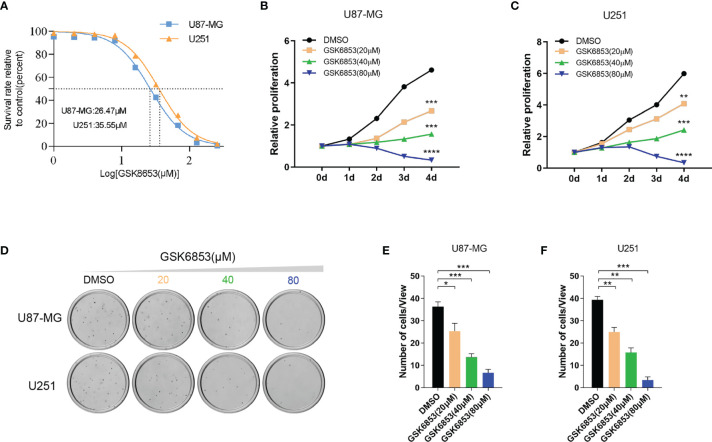
Inhibition of BRPF1 function attenuated glioma cell proliferation and colony formation. **(A)** IC50 curve of GSK6853 in U251 and U87-MG cells. Proliferation of U87-MG **(B)** and U251 **(C)** cells treated with DMSO or GSK6853 was tested by CCK-8 assays. Colony formation assay of U87-MG and U251 cells treated with DMSO or GSK6853 for three weeks **(D)** and the number of cell clones **(E, F)**. *p < 0.05; **p < 0.01; ***p < 0.001; ****p < 0.0001, two-tailed unpaired t-test.

**Figure 8:**
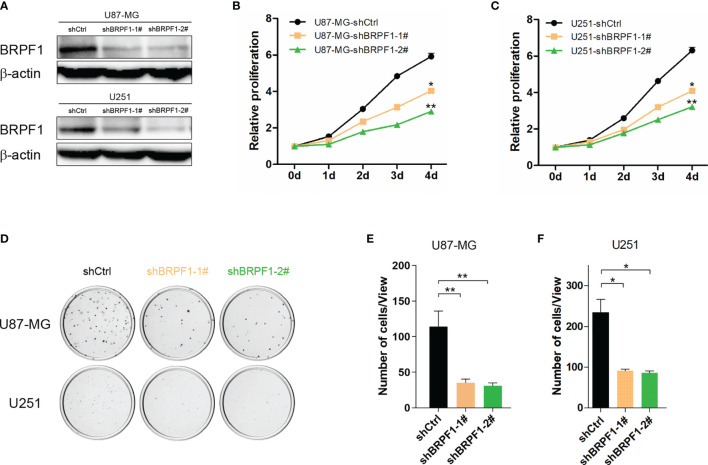
Knockdown of BRPF1 expression weakened glioma cell proliferation and colony formation. **(A)** The protein levels of BRPF1 were detected by western blot in U251 and U87-MG cells. Proliferation of U87-MG **(B)** and U251 **(C)** cells with knockdown of BRPF1 expression was tested by CCK-8 assays. Colony formation assay of U87-MG and U251 cells with knockdown of BRPF1 expression for three weeks **(D)** and the number of cell clones **(E, F)**. *p < 0.05; **p < 0.01, two-tailed unpaired t-test.

To investigate the drug target potential of BRPF1 in glioma, we selected the BRPF1-specific inhibitor GSK6853 to treat U87-MG and U251 glioma cell lines and determined the IC50 value of GSK6853 by CCK-8 assay. The results showed that GSK6853 exhibited excellent inhibitory activity against U87-MG and U251 cell lines with IC50 values of 26.47 μM and 35.55 μM, respectively (**Figure 7A**). Next, we treated U87-MG and U251 cell lines with three concentrations of GSK6853 (20 μM, 40 μM, and 80 μM) to inhibit BRPF1 function. The CCK-8 assay showed that inhibition of BRPF1 function suppressed glioma cell proliferation (**Figures 7B, C**). The number of U87-MG and U251 cell clones decreased after inhibiting BRPF1 function (**Figures 7D**–**F**). To further clarify the effect of BRPF1 expression on glioma cell proliferation, we first interfered with BRPF1 expression using shRNA in the U87-MG and U251 cell lines (**Figure 8A**). Knockdown of BRPF1 expression also attenuated the growth of U87-MG and U251 cell lines, as determined by CCK-8 assay (**Figures 8B, C**). Moreover, knockdown of BRPF1 expression reduced the clone number of U87-MG and U251 cells in the plate clone formation assay and soft agar colony formation assay (**Figures 8D**–**F**). These experiments indicated that BRPF1 is involved in glioma cell proliferation and is a potential drug target for the treatment of gliomas.

## Discussion

The survival time for LGG patients varies widely, ranging from 1 year to 15 years (41). Complete resection of LGG lesions is still a challenge because of its invasive nature, and LGG is prone to progression to glioblastoma within a few years (42). Therefore, obtaining an accurate diagnosis during the early stage of the tumor can improve the clinical outcome of patients with LGG. Inflammation is involved in tumorigenesis and progression (15–18). For example, an inflammatory microenvironment influences the growth and metastasis of gliomas (19, 20). During these processes, the expression levels of genes associated with inflammation are altered (15–17). However, the relationship between the inflammatory signature based on inflammatory response genes and the clinical outcome of patients with lower-grade gliomas remains unclear.

In this study, we first screened DEGs in primary LGG (TCGA) compared with normal brain tissue (GTEx). Thirty-five overlapping genes were obtained between the DEGs and IRGs downloaded from MSigDB. After univariate Cox regression and lasso regression analysis, nine inflammatory response genes associated with prognosis were used to construct an inflammatory signature to evaluate patient prognosis. According to the median inflammatory signature score, primary LGG in the training cohort (TCGA) and validation cohort (CGGA cohort 1 and CGGA cohort 2) was divided into high- and low-inflammatory signature groups. Subsequently, survival curves, ROC curves and risk plot distributions verified that the inflammatory signature performed well in stratifying primary LGG in the training cohort (TCGA) and validation cohort (CGGA cohort 1 and CGGA cohort 2). Furthermore, the inflammatory signature group, grade, and histology subtype were independent prognostic factors for primary LGG in the training cohort (TCGA) and validation cohort 1 (CGGA cohort 1) by multivariable Cox regression analysis. However, the inflammatory signature group was not independent prognostic factor for primary LGG in validation cohort 2 (CGGA cohort 2) by multivariable Cox regression analysis (**Figure S4B**). This occurs, in some degree, as a result of the limited number of samples in CGGA cohort 2 (n=137). The visual nomogram-based result of the multivariable Cox regression analysis was constructed and showed perfect predictive ability regarding the 1-, 3- and 5-year OS of primary LGG patients in the training cohort (TCGA) and validation cohort (CGGA cohort 1 and CGGA cohort 2).

Previous studies on inflammatory response biomarkers have mainly focused on NLR, PLR or LMR to evaluate the prognosis of patients with several types of cancers, including glioma. Cancer patients with high-inflammatory signature scores have shorter survival times (21–24). However, the quantitative values of neutrophils, lymphocytes, and platelets are derived from the peripheral blood and could not truly reflect changes in the inflammatory environment within tumor tissue. In our study, nine inflammatory response genes associated with prognosis were identified based on bulk tissue RNA-seq and clinical survival data from primary LGG. In contrast to the normal brain, SELL, TACR1, and CSF3 were downregulated while TLR3, LPAR1, ITGB8, TIMP1, MSR1, and ICAM4 were upregulated in primary LGG. Based on the mRNA expression values and coefficients from lasso regression of the nine genes, an inflammatory signature was constructed. The primary LGG patients in the high-inflammatory signature score group had a poor prognosis by Kaplan–Meier survival analysis. Univariate and multivariate Cox regression analyses showed that the inflammatory signature was an independent prognostic factor for patients with primary lower-grade gliomas. These results suggested that the inflammatory signature can not only represent the variation in the inflammatory environment inside the primary LGG but also better predict the prognosis of patients with primary LGG.

In the disease development process, changes in inflammatory levels in patients are often accompanied by an immune response (18). We first compared the difference in immune cells between the high- and low-inflammatory signature groups in the training cohort (TCGA) and validation cohort (CGGA cohort 1). The results showed that M2 macrophages were the most abundant immune cells and had a higher proportion in the high-inflammatory signature group, which indicated that M2 macrophages play a specific role in the pathogenesis of lower-grade gliomas. GO, KEGG, GSEA and GSVA analyses were also performed on DEGs between the high- and low-inflammatory signature groups. Interestingly, these DEGs were enriched in immune-related signaling pathways. These results provide further evidence that immunity participates in the pathogenesis of LGG, especially in the inflammatory response process.

During tumorigenesis and progression, gene mutation or abnormal transcription can increase gene expression and promote tumor growth and metastasis. These genes are commonly referred to as oncogenes or driver genes (43–45). However, some studies have shown that a few genes with low mutation rates may be potential driver genes of tumorigenesis. Based on the genomic transcriptome data, the NetBID algorithm was used to identify “hidden” driver genes by calculating gene activity. Genes with high activity are more likely to be potential driver genes (26). Therefore, we used the NetBID algorithm to identify 1210 driver genes between the normal brain and the high- or low-inflammatory signature group. We also screened 7102 DEGs between the normal brain and the high- or low-inflammatory signature group by bioinformatics methods. Combined with the TTD, we obtained 100 potential drug targets in the training cohort (TCGA), which were both driver genes and showed upregulated mRNA expression in gliomas. In the validation cohort (CGGA cohort 1 and CGGA cohort 2), we obtained 218 and 475 overlapping genes using same screening method, respectively. Finally, we screened out 22 common genes among TCGA cohort, CGGA cohort 1 and CGGA cohort 2. These results helped us further narrow the range of screening drug targets. Moreover, we set criteria to choose drug target gene for the following experiments validation. Firstly, the expression of target gene was higher in LGG than that in normal brain tissue. Secondly, the target gene was potential driver gene, which was calculated by NetBID algorithm in our study. Thirdly, there were small molecule inhibitors or potential clinical trial drugs for target gene. Lastly, the domain in the protein structure of target gene could be bound by small molecule inhibitors or potential clinical trial drugs.

Among 22 common genes, BRPF1 is a multivalent chromatin reader that interacts with three histone acetyltransferases, MOZ, MORF, and HBO1 (also known as KAT6A, KAT6B, and KAT7, respectively), to regulate gene expression (46–49). The forebrain-specific deletion of *Brpf1* gives rise to early postnatal lethality and growth retardation (50). Intellectual disability and facial and ocular deformities are common clinical symptoms in patients with BRPF1 mutations (51, 52). Furthermore, truncated BRPF1 was found in SHH subtype medulloblastoma (SHH-MB) in adult humans and induced SHH-MB upon SmoM2 activation in adult mice (53). Therefore, BRPF1 may play an important role in the development of the nervous system and tumorigenesis. On the other hand, BRPF1 containing a bromodomain was used for further study because it has been identified as a therapeutic target for liver cancer (40). In addition, specific inhibitors targeting bromodomain, such as JQ1, have good inhibitory effects on tumor growth (38, 39). In our study, inhibition of BRPF1 function or interference of BRPF1 expression reduced the proliferation of glioma cells *in vitro*. These results showed that BRPF1 may be a potential drug target for the treatment of gliomas.

There are several limitations that should be noted in the present study. First, due to incomplete personal clinical data, IDH mutation, MGMT methylation and 1p19q codeletion status were not included in the multivariate regression analysis to predict the outcomes of LGG patients. Second, in the analysis of gene change profiles between the high- and low-inflammatory signature groups, this study only focused on changes in gene transcription levels without considering factors such as gene mutation and methylation levels, which should be considered comprehensively. Moreover, more experiments should be performed to elucidate the underlying mechanism of BRPF1 in glioma progression and the potential of GSK6853 as a glioma target drug *in vivo*.

## Conclusion

In summary, nine inflammation-related prognostic genes were identified in primary lower-grade gliomas and applied to construct an inflammatory signature, which could be used as an independent predictor of outcomes in patients with primary lower-grade gliomas. Based on the inflammatory signature, we screened potential drug targets between the normal brain and the high- or low-inflammatory signature groups, identifying BRPF1. Inhibition of BRPF1 function attenuated glioma cell proliferation and colony formation, suggesting that BRPF1 may participate in regulating the proliferation of glioma cells. These results indicated that the inflammatory signature can be used as a candidate biomarker to predict the outcomes of patients with lower-grade gliomas and provide theoretical guidance and a decision-making basis for the clinical treatment of lower-grade gliomas.

## Data Availability Statement

Publicly available datasets were analyzed in this study. This data can be found here: University of California Santa Cruz (UCSC) Xena website (https://xena.ucsc.edu) and CGGA (http://www.cgga.org.cn).

## Author Contributions

HL, WS, and MX conceptualized, designed, and wrote the manuscript. MX, HC, TC, and PX performed data analysis and cell experiments. DM, XD, YL, and WZ revised the manuscript. All authors have read and agreed to the published version of the manuscript.

## Funding

This research was supported by grants from Children’s Hospital of Fudan University.

## Conflict of Interest

The authors declare that the research was conducted in the absence of any commercial or financial relationships that could be construed as a potential conflict of interest.

## Publisher’s Note

All claims expressed in this article are solely those of the authors and do not necessarily represent those of their affiliated organizations, or those of the publisher, the editors and the reviewers. Any product that may be evaluated in this article, or claim that may be made by its manufacturer, is not guaranteed or endorsed by the publisher.
