# Supplementary material for: Transcriptional Networks Identify BRPF1 as a Potential Drug Target Based on Inflammatory Signature in Primary Lower-Grade Gliomas
Source: Front Oncol. 2021 Dec 2;11:766656. doi: 10.3389/fonc.2021.766656 (PMC8674185; doi:10.3389/fonc.2021.766656)
Supplement: Supplementary file 1 [file DataSheet_1.pdf]

Supplementary Material

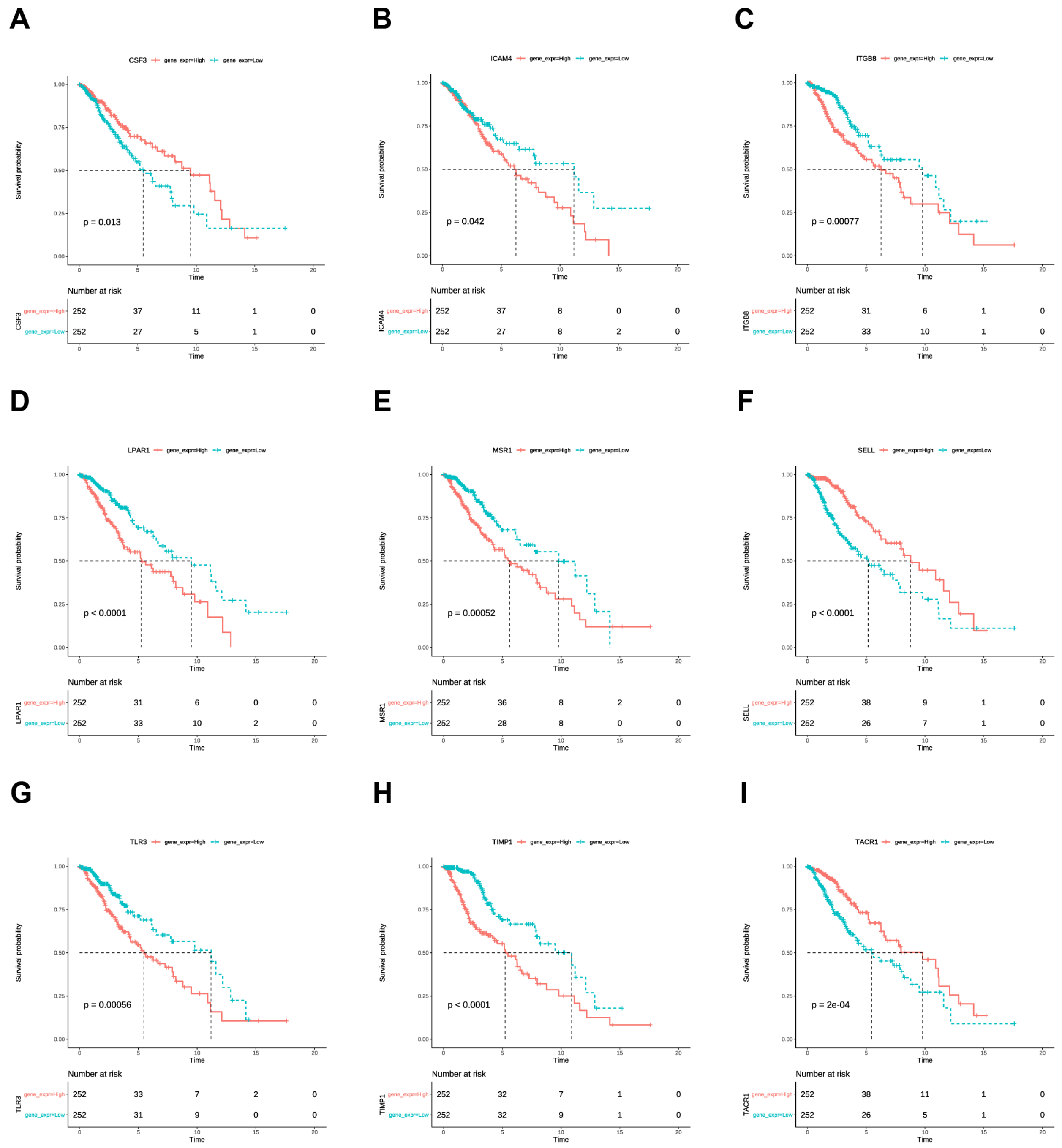

Supplementary Figure S1. The survival curve between nine genes and patients with primary LGG in TCGA cohort.

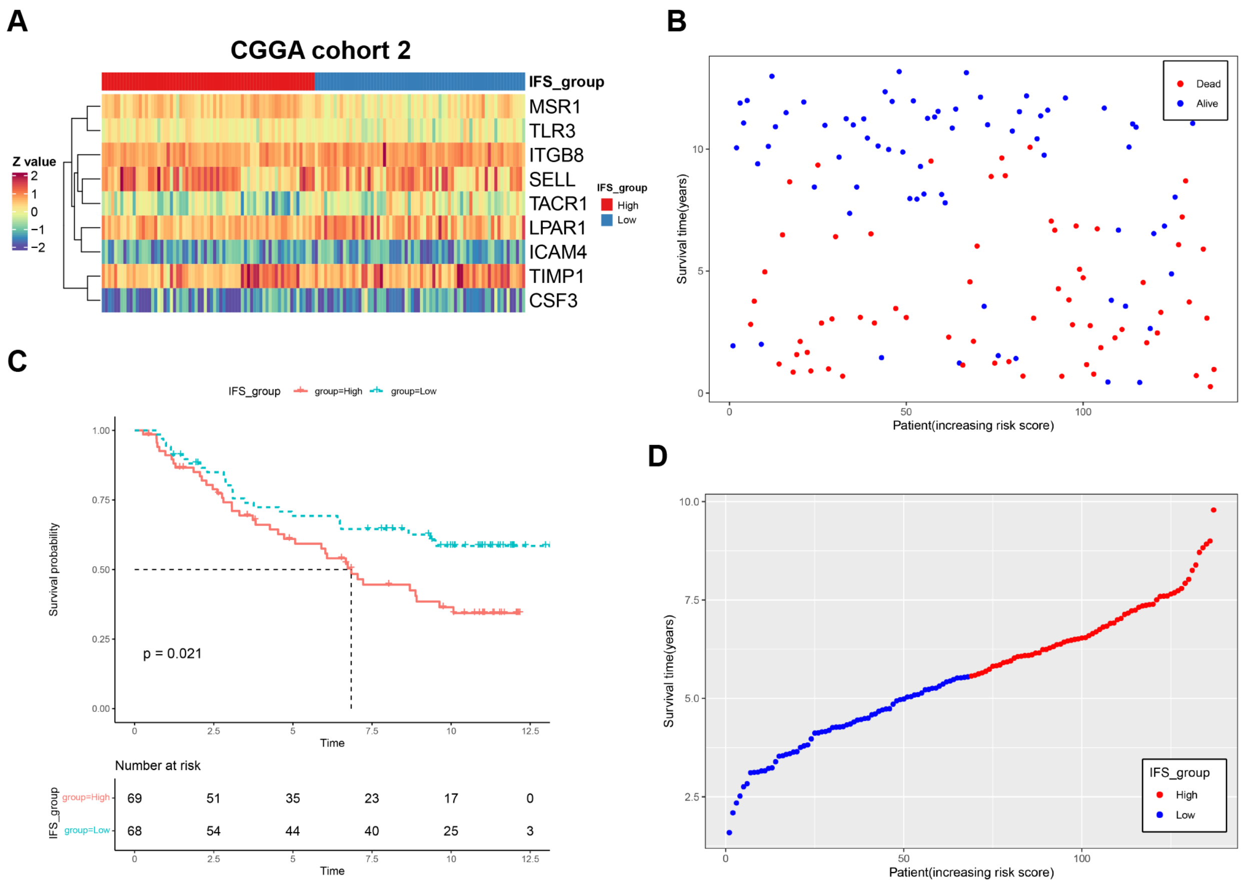

Supplementary Figure S2. LGG patients with a high inflammatory signature had a worse prognosis in the CGGA cohort 2. (**A**) Gene expression heatmap of the nine inflammation-related genes with prognostic significance in the CGGA cohort 2. (**B**) Survival status diagram of LGG patients in the CGGA cohort 2 (red dots represent death, and blue dots represent survival). (**C**) Survival curve for LGG patients with high or low inflammatory signature groups in the CGGA cohort 2. (**D**) Score distribution diagram of LGG patients with high or low inflammatory signature groups in the CGGA cohort 2 (red: high IFS; blue: low IFS).

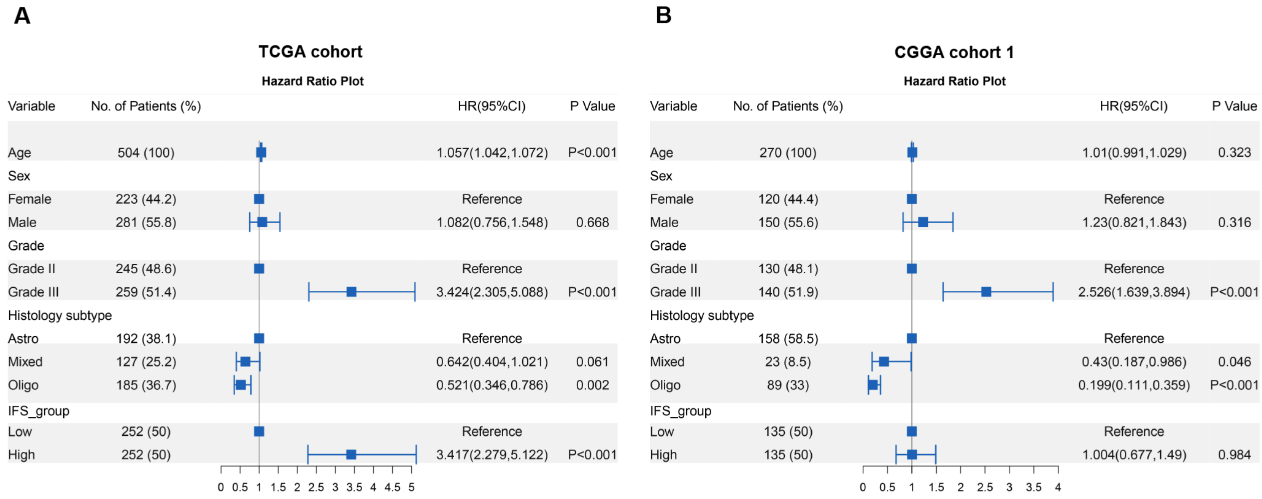

Supplementary Figure S3. Forest plot of univariate cox regression analysis in the TCGA cohort (**A**) and CGGA cohort 1 (**B**).

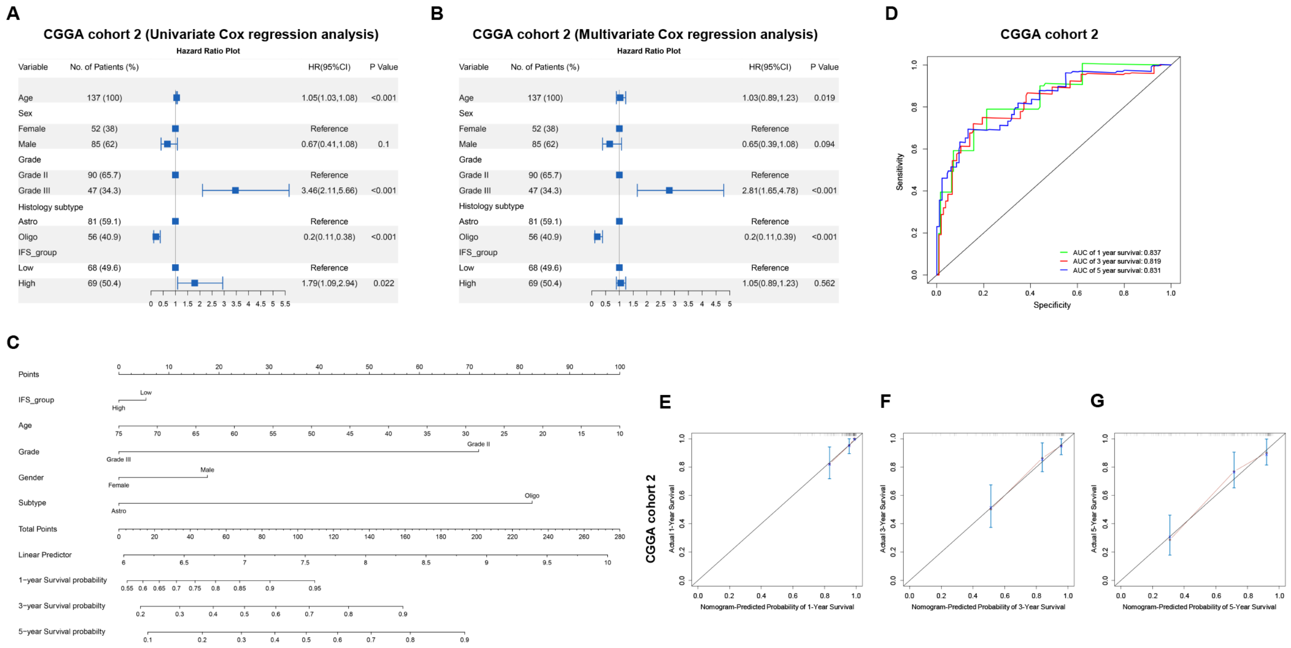

Supplementary Figure S4. The inflammatory signature was a strong prognostic factor for LGG patients in the CGGA cohort 2. (**A**) Forest plot of univariate cox regression analysis in the CGGA cohort 2. (**B**) Forest plot of multivariate cox regression analysis in the CGGA cohort 2. (**C**) Nomogram based on the results of multivariate cox regression analysis in the CGGA cohort 2. (**D**) The ROC curve and AUC of the predictions for 1, 3, and 5 years of the nomogram for the CGGA cohort 2. (**E–G**) The calibration curve for predicting the 1-, 3-, and 5-year survival in the CGGA cohort 2.

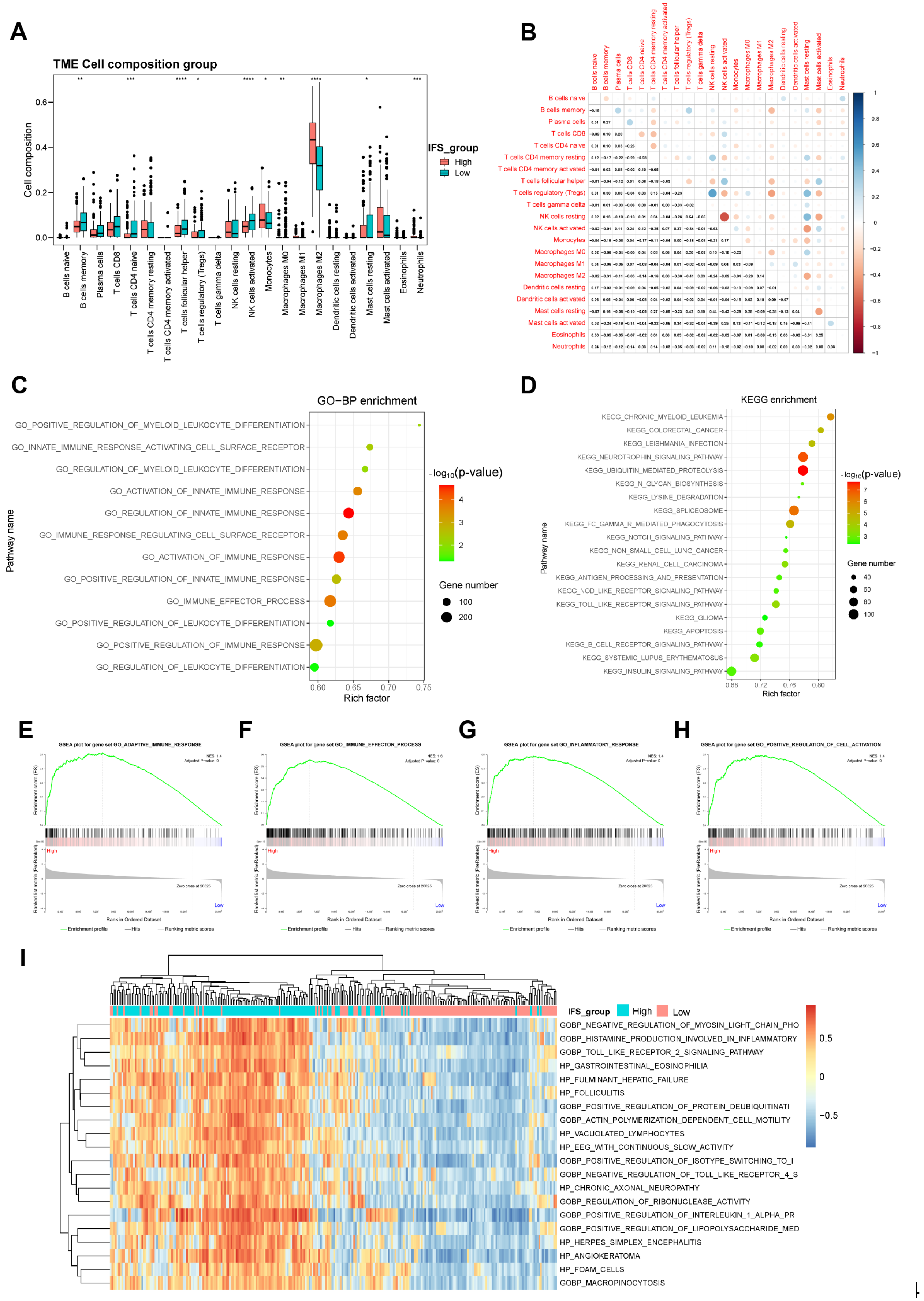
A
TME Cell composition group
Cell composition
IFS_group
High
Low
B cells naive
B cells memory
Plasma cells
T cells CD8
T cells CD4 naive
T cells CD4 memory resting
T cells CD4 memory activated
T cells follicular helper
T cells regulatory (Tregs)
T cells gamma delta
NK cells resting
NK cells activated
Monocytes
Macrophages M0
Macrophages M1
Macrophages M2
Dendritic cells resting
Dendritic cells activated
Mast cells resting
Mast cells activated
Eosinophils
Neutrophils
B
C
GO−BP enrichment
Pathway name
GO_POSITIVE_REGULATION_OF_MYELOID_LEUKOCYTE_DIFFERENTIATION
GO_INNATE_IMMUNE_RESPONSE_ACTIVATING_CELL_SURFACE_RECEPTOR
GO_REGULATION_OF_MYELOID_LEUKOCYTE_DIFFERENTIATION
GO_ACTIVATION_OF_INNATE_IMMUNE_RESPONSE
GO_REGULATION_OF_INNATE_IMMUNE_RESPONSE
GO_IMMUNE_RESPONSE_REGULATING_CELL_SURFACE_RECEPTOR
GO_ACTIVATION_OF_IMMUNE_RESPONSE
GO_POSITIVE_REGULATION_OF_INNATE_IMMUNE_RESPONSE
GO_IMMUNE_EFFECTOR_PROCESS
GO_POSITIVE_REGULATION_OF_LEUKOCYTE_DIFFERENTIATION
GO_POSITIVE_REGULATION_OF_IMMUNE_RESPONSE
GO_REGULATION_OF_LEUKOCYTE_DIFFERENTIATION
Rich factor
-log10(p-value)
Gene number
D
KEGG enrichment
KEGG_CHRONIC_MYELOID_LEUKEMIA
KEGG_COLORECTAL_CANCER
KEGG_LEISHMANIA_INFECTION
KEGG_NEUROTROPHIN_SIGNALING_PATHWAY
KEGG_UBIQUITIN_MEDIATED_PROTEOLYSIS
KEGG_N_GLYCAN_BIOSYNTHESIS
KEGG_LYSINE_DEGRADATION
KEGG_SPLICEOSOME
KEGG_FC_GAMMA_R_MEDIATED_PHAGOCYTOSIS
KEGG_NOTCH_SIGNALING_PATHWAY
KEGG_NON_SMALL_CELL_LUNG_CANCER
KEGG_RENAL_CELL_CARCINOMA
KEGG_ANTIGEN_PROCESSING_AND_PRESENTATION
KEGG_NOD_LIKE_RECEPTOR_SIGNALING_PATHWAY
KEGG_TOLL_LIKE_RECEPTOR_SIGNALING_PATHWAY
KEGG_GLIOMA
KEGG_APOPTOSIS
KEGG_B_CELL_RECEPTOR_SIGNALING_PATHWAY
KEGG_SYSTEMIC_LUPUS_ERYTHEMATOSUS
KEGG_INSULIN_SIGNALING_PATHWAY
Rich factor
E
F
G
H
I
IFS_group High Low
GOBP_NEGATIVE_REGULATION_OF_MYOSIN_LIGHT_CHAIN_PHO
GOBP_HISTAMINE_PRODUCTION_INVOLVED_IN_INFLAMMATORY
GOBP_TOLL_LIKE_RECEPTOR_2_SIGNALING_PATHWAY
HP_GASTROINTESTINAL_EOSINOPHILIA
HP_FULMINANT_HEPATIC_FAILURE
HP_FOLLICULITIS
GOBP_POSITIVE_REGULATION_OF_PROTEIN_DEUBIQUITINATI
GOBP_ACTIN_POLYMERIZATION_DEPENDENT_CELL_MOTILITY
HP_VACUOLATED_LYMPHOCYTES
HP_EEG_WITH_CONTINUOUS_SLOW_ACTIVITY
GOBP_POSITIVE_REGULATION_OF_ISOTYPE_SWITCHING_TO_I
GOBP_NEGATIVE_REGULATION_OF_TOLL_LIKE_RECEPTOR_4_S
HP_CHRONIC_AXONAL_NEUROPATHY
GOBP_REGULATION_OF_RIBONUCLEASE_ACTIVITY
GOBP_POSITIVE_REGULATION_OF_INTERLEUKIN_1_ALPHA_PR
GOBP_POSITIVE_REGULATION_OF_LIPOPOLYSACCHARIDE_MED
HP_HERPES_SIMPLEX_ENCEPHALITIS
HP_ANGIOKERATOMA
HP_FOAM_CELLS
GOBP_MACROPINOCYTOSIS

Supplementary Figure S5. Identification of the immune cell landscape and transcriptional characteristics between the high and low inflammatory signature groups in the CGGA cohort 1. (**A**) Comparison of the immune cell composition between the high and low inflammatory signature groups in the CGGA cohort 1. (**B**) Correlation of 22 types of immune cell subsets in the CGGA cohort 1. GO (**C**), KEGG pathway (**D**), GSEA (**E-H**) and GSVA (**I**) analyses of differentially expressed genes between the high and low inflammatory signature groups in the CGGA cohort 1. *p < 0.05; **p < 0.01; ***p < 0.001; ****p < 0.0001; Wilcoxon test was used to assess the significance of the immune cell composition.
